# Structural, Morphological, Electronic Structural, Optical, and Magnetic Properties of ZnO Nanostructures

**DOI:** 10.3390/ma15248889

**Published:** 2022-12-13

**Authors:** Nisrin Alnaim, Shalendra Kumar, Adil Alshoaibi

**Affiliations:** 1 Department of Physics, College of Science, King Faisal University, P.O. Box 400, Al-Ahsa 31982, Saudi Arabia; 2 Department of Physics, University of Petroleum & Energy Studies, Dehradun 248007, India

**Keywords:** ZnO nanowires, vapor–liquid–solid (VLS) method, photoluminescence (PL), Raman spectroscopy, ferromagnetic (FM)

## Abstract

ZnO nanostructures were grown on a Si(111) substrate using a vapor–liquid–solid (VLS) growth procedure (pristine ZnO) and annealed via a rapid thermal-annealing process in an argon atmosphere at 1100 °C (Ar-ZnO). The synthesized ZnO nanostructures were investigated through structural, electronic structural, morphological, optical, and magnetic characterizations. X-ray diffraction and selective area electron diffraction (SAED) measurements revealed that both samples exhibited the hexagonal wurtzite phase of nanocrystalline ZnO. Near-edge X-ray absorption fine structure (NEXAFS) spectroscopy carried out at the O K-edge inferred the presence of the intrinsic-defect states. Field-emission scanning electron microscopy (FE-SEM) and transmission electron microscopy images displayed the formation of ZnO nanostructures. The photoluminescence (PL) spectra demonstrated an emission band in the UV region along with an additional defect band in the visible region. PL spectral analysis confirmed the presence of intrinsic defects in Ar-ZnO nanowires, contributing to the enhanced emission in the visible region. The Raman spectra showed the characteristic band (434 cm^−1^) corresponding to the vibrational modes of hexagonal wurtzite ZnO, with an additional band attributable to intrinsic defects. DC magnetization measurements showed a ferromagnetic response in both samples with enhanced coercivity in Ar-ZnO (~280 Oe). In brief, both samples exhibited the presence of intrinsic defects, which are found to be further enhanced in the case of Ar-ZnO. Therefore, it is suggested that intrinsic defects have played an important role in modifying the optical and magnetic properties of ZnO with enhanced results for Ar-ZnO.

## 1. Introduction

The n-type direct wide-bandgap semiconductor (3.37 eV), ZnO, has been highly investigated by researchers to utilize it in the manufacturing of semiconductor-based devices, such as solar cells and gas sensors, mainly due to its high abundance, environmental compatibility, and low cost [1,2]. A high room-temperature exciton-binding energy of ~60 meV and high electron mobility of ZnO makes it useful for LED and electron-transporting material (ETM) applications, respectively [1,3,4]. In addition to that, undoped ZnO displays traces of magnetism, which can make it useful in spintronic-based applications, such as enhanced data storage capacity and processing speed [5,6]. Owing to the coexistence of manifold properties, the interaction between electronic, optical, and magnetic assets allow ZnO to be a potential replacement for currently used semiconductors, such as GaAs, GaN, etc., for which the highest reported Curie temperature is below room temperature [7]. Therefore, it is paramount to enhance the optical, electronic, and magnetic properties in order to manufacture highly efficient devices [8]. Lukasz Kuna et al. have shown through finite-element-method-based modeling that the change in shape and size of nanowires can modulate the optical properties of the material to significant levels [9]. One-dimensional nanostructures, such as nanowires, nanostructures, etc., have been reported to be important in making interconnections and functional components in the fabrication of technological devices [10]. Effectively, the morphological evolution of ZnO along one dimension in the form of nanowires (NWs) introduces strong confinement effects by restricting the motion of charge carriers and photons along one direction only, which may lead to the development of desirable properties [1]. The dynamical tuning of electronic properties in nanowires can also be useful for energy harvesting [9]. Alongside this, the highly anisotropic wurtzite structure of ZnO favors the easy growth of NWs [4]. The methods employed to grow NWs are equally important while influencing the properties of the material [11]. Various methods have been used to prepare ZnO nanowires utilized for various applications. For instance, Navarrete et al. grew ZnO nanowires on alumina substrates using a chemical-vapor-deposition technique for gas-sensing (ethanol and N_2_) applications [12]. Likewise, Guan et al. grew an array of ZnO nanowires using the wet-chemical method, which worked as a self-powered ultraviolet photodetector sensor [13]. Copper-decorated ZnO nanowires were prepared using chemical vapor deposition and magnetron-sputtering techniques by Han et al. to modify the photoelectrochemical (PEC) properties [14]. These reports are indicative of the fact that the highly adaptable nature of ZnO makes the tuning of its properties favorable for functional applications due to the structural versatility of ZnO. Interestingly, pure ZnO exhibits many types of intrinsic defects, such as oxygen vacancies (O_v_), oxygen interstitials (O_i_), zinc vacancies (Zn_v_), and zinc interstitials (Zn_i_), without deviating from its stoichiometry. The presence of defects enhances the chemical reactivity of ZnO, which yields a convenient interplay between various properties.

In the present work, the vapor–liquid–solid (VLS) technique has been utilized for the growth of ZnO nanostructures. VLS is a foreign element catalytic agent (FECA)-mediated method and was initially proposed by Wagner in the 1960s [10]. It is a widely adopted method for the synthesis of nanowires [15] with the advantage of providing the controlled synthesis of the ordered arrays of nanowires with desirable diameters [16]. As-prepared and Ar-annealed ZnO nanostructures have been investigated using various characterizations, such as X-ray diffraction (XRD), NEXAFS spectroscopy, field-emission transmission electron microscopy (FE-SEM), photoluminescence (PL) spectroscopy, Raman spectroscopy, and DC magnetization. The XRD, SAED, and Raman spectroscopy results demonstrate that as-prepared and Ar-annealed ZnO nanostructures exhibit the single-phase wurtzite crystal structure. O K-edge measurements reveal an increase in defects in Ar-annealed ZnO nanostructures. The PL spectroscopy measurements displayed NBE, along with emission in the visible region. The DC magnetization results indicate that ZnO nanostructures have ferromagnetic ordering at room temperature.

## 2. Experimental

ZnO nanowires were grown using the vapor–liquid–solid (VLS) method. In the typical synthesis, commercial ZnO powder (purity 99.999%, Sigma-Aldrich, St. Louis, MO, USA) was kept in the tube furnace along with the silicon substrate, which was placed 10.0 cm away from the ZnO powder. The powder was allowed to evaporate at 1100 °C for 30 min. Finally, ZnO nanostructures were grown on the Si(100) substrate. Two such samples were prepared in similar conditions. The second sample was further annealed using rapid thermal annealing at 1100 °C for 120 s in ambient argon (Ar) gas using the ULVAC (QHC, VHC-P610, Yokohama, Japan) system. The heating and cooling rate of the RTA system was kept at 35 °C/s throughout the process of annealing. The synthesized sample was named pristine ZnO, while the sample annealed in the presence of Ar was assigned Ar-ZnO. The ZnO nanostructures were characterized using X-ray diffraction (XRD), near-edge X-ray absorption fine structure (NEXAFS) spectroscopy at the O K-edge, field-emission scanning electron microscopy (FE-SEM), photoluminescence (PL), Raman spectroscopy, and DC magnetization. Phillips X’pert (MPD 3040, EA, Almelo, Netherland) with a Cu Kα source (λ = 1.54 Å) diffractometer was utilized for crystal structure analysis. The O K-edge spectra were measured with the soft X-ray beamline 10D XAS KIST (Korea Institute of Science and Technology) of Pohang Accelerator Laboratory (PAL). The surface morphology of the ZnO nanostructures was captured by a field-emission electron microscope (FESEM, JSM-7500, JEOL, Tokyo, Japan). The high-resolution transmission electron microscopy (HR-TEM) images and selected-area electron diffraction (SAED) pattern were recorded using an FE-TEM (JEM-2100 F, Corporation Place, Singapore) operating at 200 kV. The PL spectra were measured using the Lab RAM HR Evolution Horiba single monochromator using a 325 nm helium–cadmium laser as an excitation source. A Raman spectrometer (NRS-3100) (S-4100) of SINCO Instrument Co. (Seoul, Republic of Korea) was also used. Magnetic properties were studied using a commercial Quantum Design physical properties measurement system (PPMS).

## 3. Results and Discussion

### 3.1. X-ray Diffraction (XRD)

**Figure 1:**
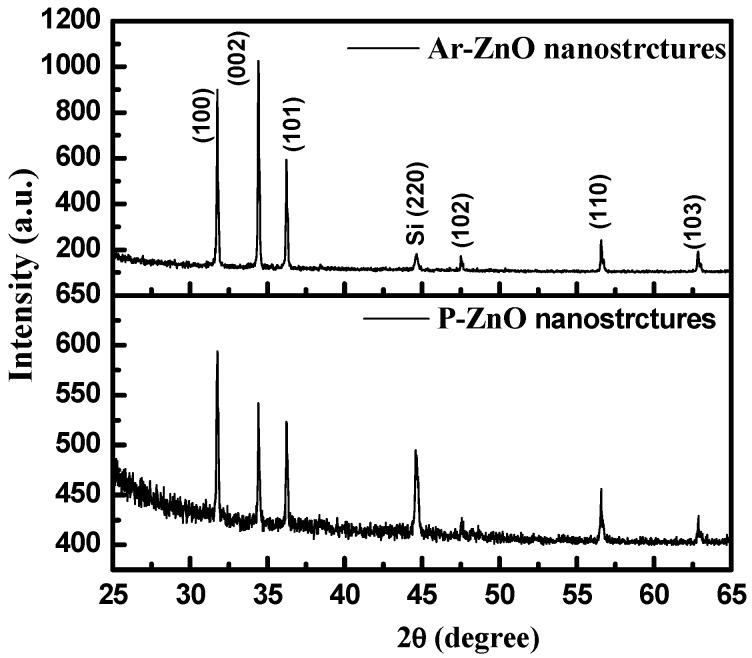
XRD patterns for pristine ZnO and Ar-ZnO nanowires grown on Si(100) substrate.

The X-ray diffraction patterns of the pristine ZnO and Ar-ZnO nanostructures grown on the Si substrate measured in θ-2θ are displayed in Figure 1. The sharp peaks in the XRD patterns are attributable to the hexagonal wurtzite phase of nanocrystalline ZnO with planes assigned (100), (002), (101), (102), (110), (103), (200), (112), (004), and (202) in accordance with the JCPDS card number (036-1451) [2]. The additional peak corresponds to the Si (220) substrate [17]. Apart from these, no extra peaks were detected, which rules out the presence of any kind of impurities. No noticeable shift in the peak positions was observed, indicating that the lattice parameters and, hence, the volume remained the same in both samples. The values of the lattice parameters were found to be a (=b) = 3.20 Å and c = 5.20 Å, which is in agreement with previously reported values [18].

### 3.2. O K-Edge Spectroscopy

**Figure 2:**
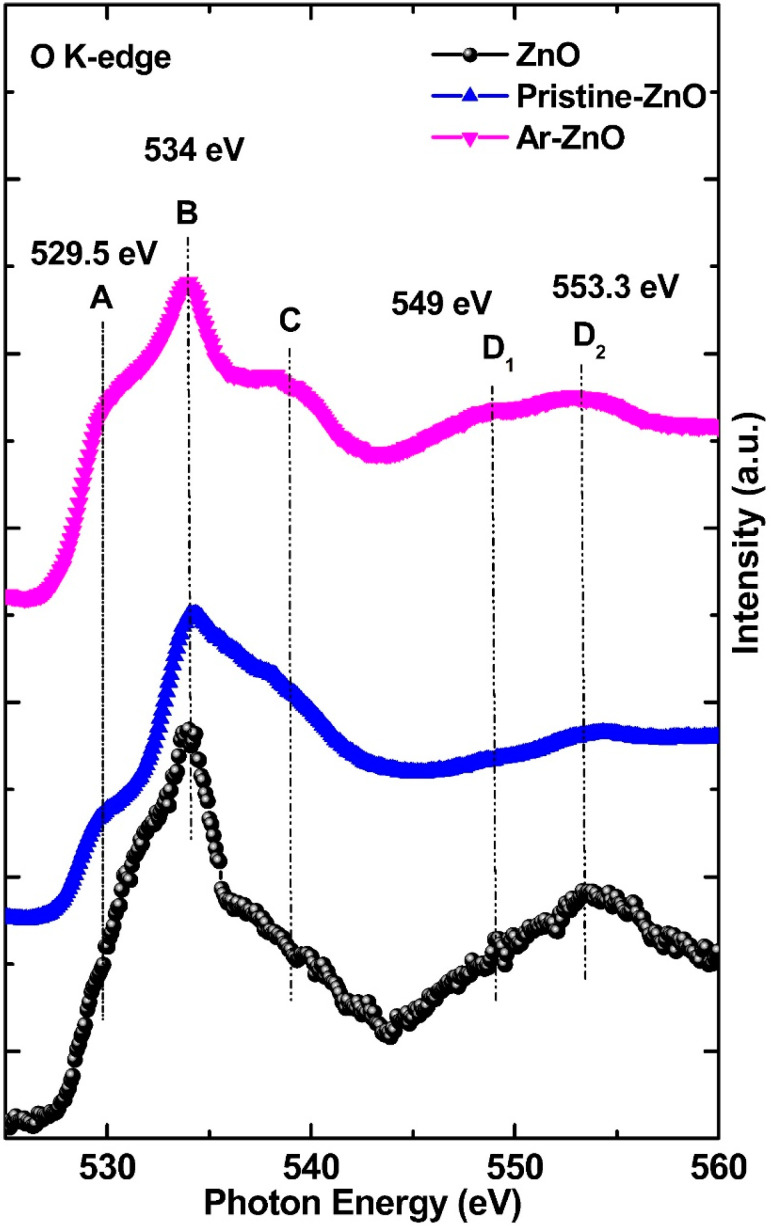
O K-edge spectra in the energy range of 525–560 eV of pristine ZnO and Ar-ZnO, along with standard ZnO.

X-ray absorption spectroscopy is an important tool used to investigate the local electronic structure of the probed ion [19,20]. Oxygen is very important in the determination of the chemical reactivity of ZnO. Therefore, it is important to investigate the oxidation state of the O-atom in ZnO. The spectral fingerprint obtained when O 1s electron is excited to the lowest unoccupied level after absorbing X-rays is known as the O K-edge spectra. The electronic transitions occurring from O 1s to O 2p states, resulting in the O K-edge spectral features, are in agreement with dipole-selection rules [21]. The O K-edge spectra are generally broad and provide information about the oxidation state acquired by the O-atom in the material [22]. The O K-edge spectra are displayed in Figure 2 in the energy range of 520–560 eV. Because Zn 3d orbitals are all filled, hybridization takes place between O 2p and Zn 4sp, resulting in peak splitting. The spectra show the O K-edge transitions of pristine ZnO and Ar-ZnO, along with the standard ZnO: O K-edge spectra. The main peak was obtained at 534 eV, which is in agreement with standard ZnO spectra, as well as previously reported work [23,24]. Along the lower energy side of the main peak, a shallow shoulder was accompanied at 529.5 eV. The appearance of this shoulder peak in undoped ZnO is interesting because it typically emerges due to electronic transitions from 2p to 3d states; however, 3d states were all occupied in the case of undoped ZnO. Therefore, this shoulder peak should ideally not be observed as it can also be compared with standard ZnO spectra. Therefore, the presence of a shoulder peak at 529.5 eV suggests that some 3d states may have been available (perhaps due to the change in the oxidation state of Zn) and resulted in a few O 2p–Zn 3d transitions. Likewise, on the higher energy side of the main peak, another shoulder was observed. which is not notable in standard ZnO spectra. Overall, a small change in the spectral features is clearly visible; although, all the features in Ar-ZnO were more enhanced, along with the main peak at 534 eV, compared to pristine ZnO, pointing towards the presence of sharp transitions in Ar-ZnO. These transitions point towards the presence of intrinsic defects which are enhanced in Ar-ZnO compared to pristine ZnO. Furthermore, in the observed spectra, the peak positions were not found to change; however, the intensity of Ar-ZnO was smaller than pristine-ZnO, meaning there was a decrease in the unoccupied states as the intensity is a measure of the unoccupied states.

### 3.3. Morphological Analysis

**Figure 3:**
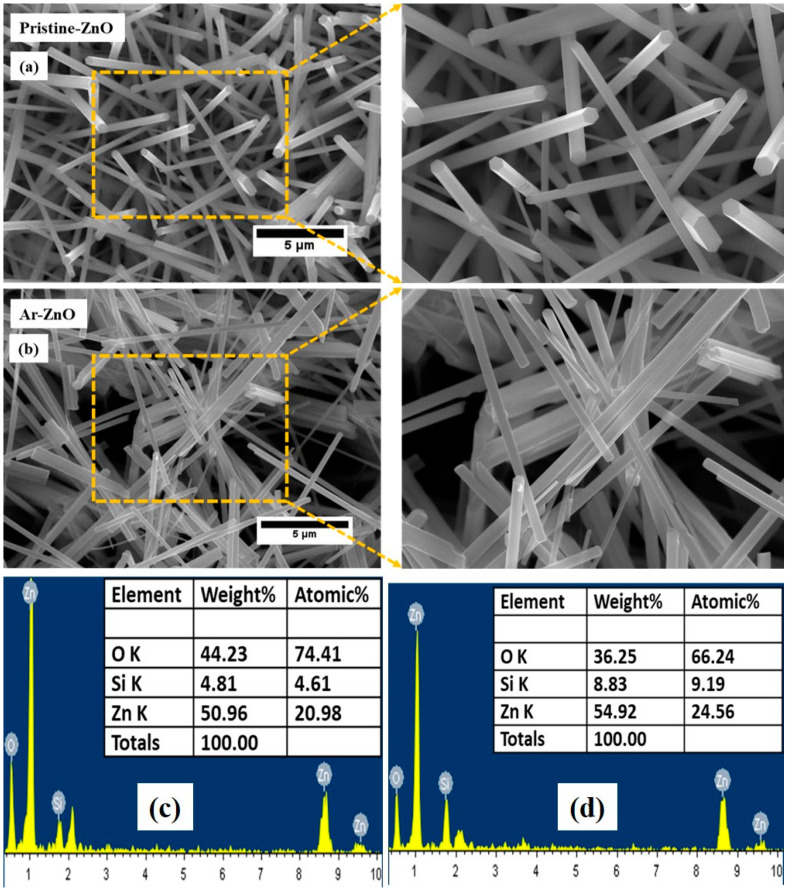
(**a**,**b**) The FESEM images of pristine ZnO and Ar-ZnO nanowires, respectively, along with the enlarged view of one portion of the images, (**c**,**d**) EDX spectra of pristine ZnO and Ar-ZnO nanowires.

**Figure 4:**
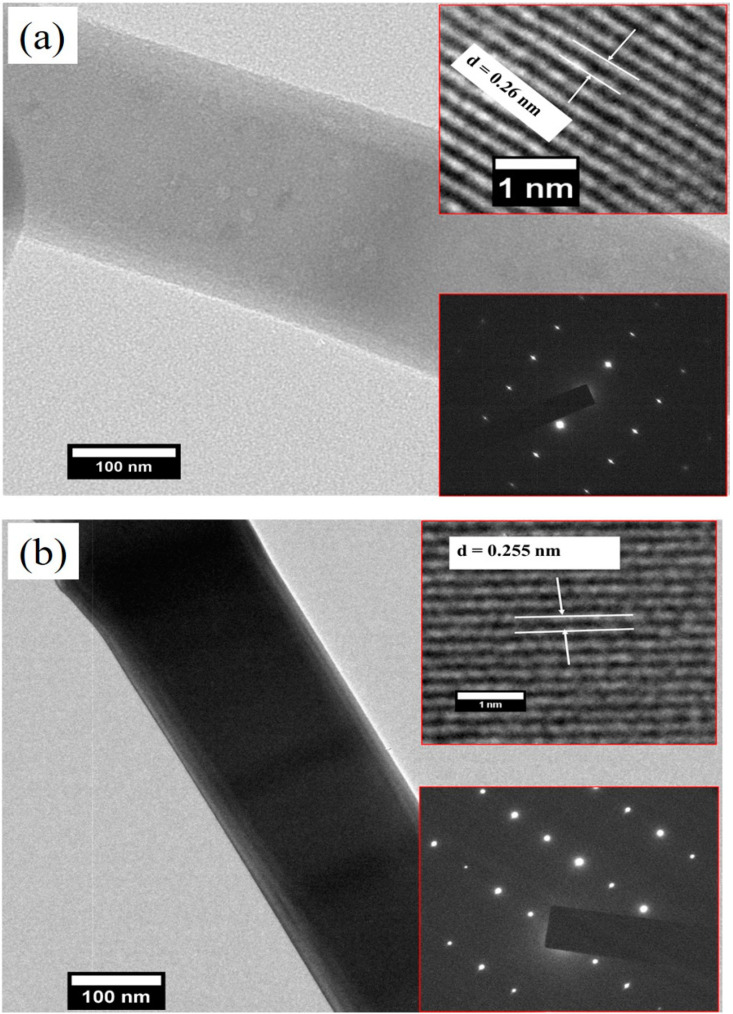
(**a**,**b**) TEM micrographs of pristine ZnO and Ar-ZnO nanowires. Inset shows the SAED pattern and HR-TEM image of pristine ZnO and Ar-ZnO nanowires.

Figure 3a,b present the HR-FESEM images of the pristine ZnO and Ar-ZnO nanowires, respectively. The excellent manifestation of the nanostructures is clearly evident. The random orientation of the nanowires may be due to the chemical route procedure opted for in the preparation of the nanostructures. The primary inspection of the nanowires indicated that the presence of the argon atmosphere noticeably improved the quality of the nanowires. The nanowire dimensions were determined using the Image-J software. It was observed that the diameter of the P-ZnO and Ar-ZnO nanostructures were 386.0 nm and 268 nm, respectively. Here, it is worth mentioning that it is difficult to determine the exact length of the ZnO nanostructure; however, the rough estimation of the aspect ratio calculated from FE-SEM micrographs indicates that both samples have nanocrystalline morphology. The energy dispersive X-ray spectroscopy (EDX) measurements of the P-ZnO and Ar-ZnO nanostructures are highlighted in Figure 3c,d. The EDX measurements inferred the presence of Zn and O elements only and ruled out any impurities. Figure 4a,b reveals the HR-TEM micrograph and SAED pattern of the P-ZnO and Ar-ZnO nanostructures. The TEM images conclude that the ZnO nanostructures exhibited a rod-like nanostructure and agreed with the FE-SEM results. The indexing of the SAED pattern further supports the single-phase nature of the ZnO nanostructures. The HR-TEM micrograph was used to determine the interplanar distance and found 0.25 nm, which is consistent with the ZnO (002) plane.

**Figure 5:**
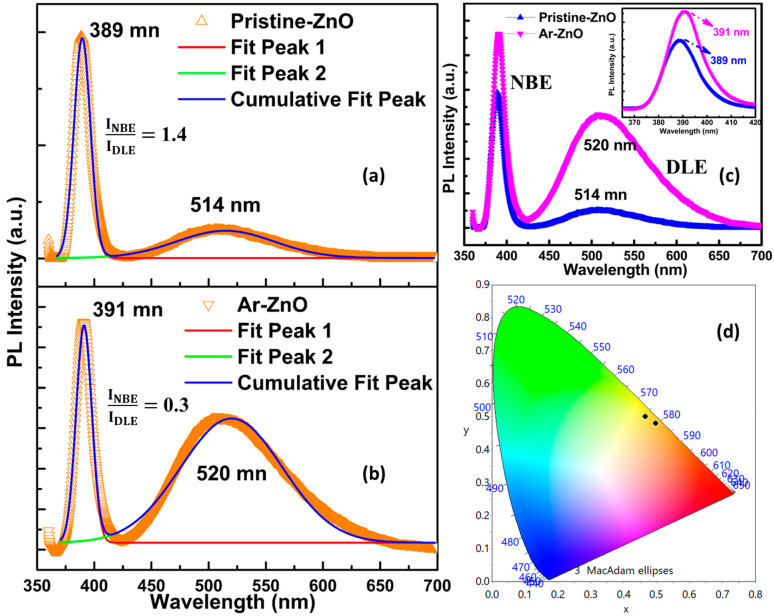
(**a**,**b**) Fitted PL spectra of pristine ZnO and Ar-ZnO nanowires, respectively, along with the ratio of NBE to DLE; (**c**) Comparative display of pristine ZnO and Ar-ZnO spectra; and (**d**) CIE 1931 chromaticity diagram showing the color of emitted light.

Figure 5a–d shows the PL spectra of the samples in UV and the visible region obtained at room temperature. The room-temperature photoluminescence of ZnO may be associated with its sufficiently large exciton-binding energy (60 meV). The fitting of the spectra (Figure 5a,b) showed two well-observed bands in the range of 350–700 nm. Both the samples exhibited the main strong band in the UV region at 389 nm and 391 nm, which is characteristic of ZnO when its dimensions are reduced in the nanoscale region. The position of the band in the UV region lies in the range of 370–390 nm, depending on the morphology [25]. Therefore, the band shift in the present case also suggests an improved morphology and aspect ratio of the nanowires, as has been observed in the HR-FESEM images. This strong and narrow band in the UV region is referred to as the near-band-edge emission (NBE) as it resulted from the band-edge/excitonic recombination, which occurs at discrete electronic states created by the complex within the bandgap region [11]. The other band was observed in the visible region at 514 nm and 520 nm for pristine ZnO and Ar-ZnO, respectively, indicating the green-colored exciton luminescence. The relatively smaller band observed in the visible region is, generally, the result of the defect-induced excitations allowed within the band gap; therefore, it is described as deep-level emission (DLE). It is clear from Figure 5a,b that DLE bands are very broad, ranging from 425 nm to 650 nm, which indicates that the emissions were the result of the superposition of the recombinations of many deep-level transitions involving emission in the blue, green, yellow, and orange regions. The origin of the DLE emission of the ZnO may be associated with intrinsic vacancies, such as oxygen vacancies (O_v_), oxygen interstitials (O_i_), zinc vacancies (Zn_v_), and zinc interstitials (Zn_i_) [26]. The charge states of these defects are an important factor in determining the nature of allowed transitions. The color of the emitted radiation depends on the type of defect taking part in the transition. For instance, the blue emission is associated with Zn_v_, while the green emission may be attributed to Ov or Zn_i_. Similarly, the yellow emission comes from the O_i_. Thus, the broad bands observed in the present case were the result of radiative transitions involving various types of native defects [11]. The enhancement in the DLE band in the Ar-ZnO in comparison with the pristine ZnO may be interpreted as an increase in the number of native defects in the Ar-ZnO nanowires. Further, the intensity of the UV emission strongly depends on the crystallinity of ZnO. Therefore, the intensity NBE band in Ar-ZnO was increased to a noticeable amount (see Figure 5c) compared to that of pristine ZnO. The FWHM of the NBE band was also found to be reduced in Ar-ZnO which is another indication of improved crystallinity [27,28]. In addition to the crystallinity, the surface-induced recombinations also added to the intensity of the NBE bands because the decrease in the diameter and the increase in the length of the nanowires may lead to an increase in the surface-to-volume ratio due to which the surface states also primarily take part in the transitions [11]. Thus, increasing intensity revealed an increase in the concentration of the intrinsic defects in the Ar-ZnO nanowires [27]. The ratio of the integrated spectra intensity of NBE to DLE bands yielded the contribution of the defect states to the photoluminescence emission of the material, which was 1.4 and 0.3 for pristine ZnO and Ar-ZnO, respectively, in the present case. The highest intensity represents the lowest defect states in the material. Thereby, the lower NBE/DLE ratio for Ar-ZnO represents the higher defects compared to pristine ZnO. Further, the optical response was also investigated using the chromaticity diagram obtained using Color Calculator v7.77. Moreover, the redshift observed in the Ar-ZnO nanowires was also visible in the CIE diagram as shown in Figure 5d. The parameters of CIE analysis, i.e., the XY coordinates, correlated color temperature (CCT), color rendering index (CRI), and luminous efficacy of radiation (LER), for pristine ZnO and Ar-ZnO are displayed in Table 1. This also reveals the increase in the chromaticity rendering index (CRI) of the Ar-ZnO nanowires.

### 3.4. Raman Spectroscopy

**Figure 6:**
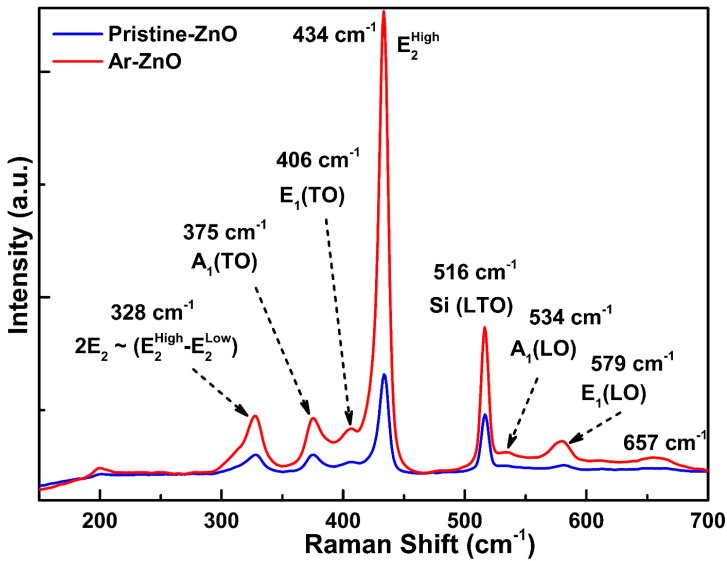
Raman spectra of pristine ZnO and Ar-ZnO nanowires.

The Raman spectra of the ZnO nanowires grown on the Si substrate as pristine and in the Ar atmosphere are displayed in Figure 6 in the range of 200–700 cm^−1^. The Raman phonon mode corresponding to Si was observed at 516 cm^−1^. The remaining Raman active modes are attributed to ZnO. The highest intensity mode observed at 434 cm^−1^ is ascribed to the non-polar phonon active mode of ZnO denoted by $E_{2}^{\mathrm{High}}$ [27]. The highly intense $E_{2}^{\mathrm{High}}$ mode corresponds to the characteristic band of the hexagonal wurtzite structure of ZnO, indicating Zn-O vibrations. Zhang et al. reported the $E_{2}^{\mathrm{High}}$ vibrational mode at 437.2 cm^−1^. The intensity, as well as the full width at half maximum (FWHM), increased in Ar-ZnO compared to pristine ZnO. The increase in the FWHM directly indicates the presence of defects. The nature of defects may be intrinsic as analyzed through the PL spectra. The other bands observed at 375, 406, 534, and 579 cm^−1^ are associated with the polar transverse-optical (TO) and longitudinal-optical (LO) first-order vibrational modes denoted as A_1_(TO), E_1_(TO), A_1_(LO), and E_1_(LO), respectively. Another vibrational mode was observed at 657 cm^−1^, which is attributed to the intrinsic defects. Evidently, the intensity of the defect vibrational band was higher in the Ar-ZnO nanowires which imply enhanced defects in the Ar-ZnO nanowires. Additionally, the observation of defects is in agreement with those observed in PL spectral investigations. The vibrational mode observed at the lower frequency of 328 cm^−1^ corresponds to second-order vibrations [29]. Thus, the use of an Ar atmosphere affects Raman scattering, demonstrating not only the enhanced vibrational modes of undoped Zn-O, but also the enhanced intrinsic-defects mode.

### 3.5. Magnetization Analysis

**Figure 7:**
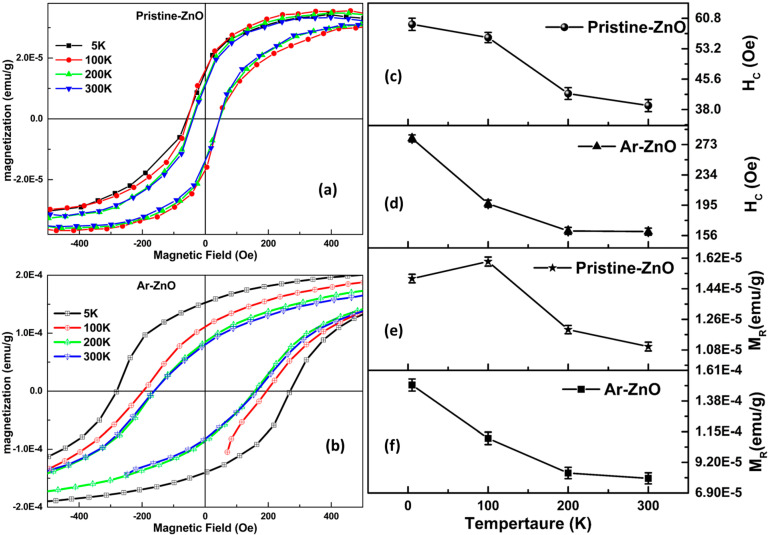
(**a**,**b**) Magnetization (M) vs. magnetic field (H) hysteresis loops of pristine ZnO and Ar-ZnO nanowires, respectively; (**c**–**f**) Variation of coercivity (Hc) and remnant magnetization (M_R_) with temperature from 5 K–300 K.

The magnetic-field-dependent magnetization curves, measured at 5 K, 100 K, 200 K, and 300 K, are displayed in Figure 7a,b for pristine ZnO and Ar-ZnO, respectively. Although both curves show ferromagnetic behavior, the effect of the Ar atmosphere can be clearly observed on the hysteresis. After subtracting the diamagnetic contribution, the saturation magnetization was found to be decreased in the case of the Ar-ZnO nanowires. However, the hysteresis in Ar-ZnO increased significantly compared to that of pristine ZnO. The effect of temperature was also prominent in the case of Ar-ZnO. With increasing temperature, the coercivity and remanence were found to decrease and become minimal at room temperature. The decreasing trend in coercivity and remanence can be observed in Figure 7c–f. Further, the origin of the ferromagnetic behavior in undoped ZnO may be associated with intrinsic defects. However, the highly sought origin of ferromagnetic character in undoped ZnO remains controversial. Nevertheless, various models and theories have been proposed in order to understand the fundamental mechanisms, such as the Zener model, bound magnetic polaron model (BMP), mean-field theory, etc. [30]. Undoped ZnO has been studied extensively by various research groups. The presence of ferromagnetic response may be associated with intrinsic or extrinsic sources. Intrinsic sources may be intrinsic/native defects, while extrinsic sources may be the secondary magnetic phase or magnetic clusters. Kumar et al. associated the presence of a ferromagnetic signature in ZnO nanostructures to Zn vacancies (Zn_V_) [5]. Thus, the induced ferromagnetism in undoped ZnO has been associated with intrinsic defects [31,32]. The possibility of extrinsic sources can be easily ruled out in undoped ZnO. On the other hand, intrinsic defects have become very customary, especially when the chemical route is employed for the synthesis of undoped ZnO. In our case, we have confirmed the presence of intrinsic defects in both the samples of pristine ZnO and Ar-ZnO. Nonetheless, the defects were found to be increasing in Ar-ZnO, which has improved the morphological, optical, and magnetic various properties of ZnO.

## 4. Conclusions

In summary, the controlled growth of ZnO nanowires has been achieved using the vapor–liquid–solid method. The structural, electronic structural, morphological, optical, and magnetic properties of the synthesized nanowires were investigated through XRD, O-K-edge spectroscopy, HR-SEM, HR-TEM, photoluminescence emission spectra, Raman spectroscopy, and DC magnetization. The HR-TEM, SAED, and XRD patterns confirmed the hexagonal wurtzite phase of ZnO. The electronic transitions, other than the O K-edge, were also observed in the O K spectra, which indicates the possible presence of defect states in the samples. The rod-type morphology of the nanostructure was observed in the TEM and FE-SEM images and showed an enhanced aspect ratio for Ar-ZnO nanowires. The optical properties were studied through photoluminescence (PL) emission spectra as well as Raman spectroscopy. Both these spectra showed the characteristic bands corresponding to nanocrystalline ZnO, along with the intrinsic-defect bands. However, the response of the PL emission and Raman bands was found to be enhanced for Ar-ZnO nanowires compared to the pristine ZnO nanowires. In addition, both samples showed ferromagnetic behavior; however, the coercivity of Ar-ZnO nanowires was found to be increased. The enhanced optical and ferromagnetic response of Ar-ZnO nanowires may be associated with intrinsic defects as the presence of intrinsic defects has been confirmed using various characterizations.

## Figures and Tables

**Table 1:** The values of XY coordinates, correlated color temperature (CCT), color rendering index (CRI), and luminous efficacy of radiation (LER) for pristine ZnO and Ar ZnO.

Samples	x	y	CCT	CRI	LER
ZnO-Pristine	0.4670	0.5000	3210	58	386
ZnO-Ar	0.4981	0.4783	2695	63	346

## Data Availability

Available on request.
